# Study on synthesis of ursodeoxycholic acid by reduction of 7-ketolithocholic acid in double aprotic solvents and molecular simulations

**DOI:** 10.1186/s40643-023-00668-x

**Published:** 2023-08-07

**Authors:** Mohan Dai, Binpeng Xu, Qing Guo, Junfen Wan, Xuejun Cao

**Affiliations:** State Key Laboratory of Bioreactor Engineering, Department of Bioengineering, East China University of Science and Technology, Shanghai, 200237 China

**Keywords:** 7-Ketolithocholic acid, Ursodeoxycholic acid, Aprotic solvent, Linear sweep voltammetry, Molecular simulation

## Abstract

Ursodeoxycholic acid (UDCA) is not only safer than chenodeoxycholic acid in the treatment of hepatobiliary diseases, but also has a wide range of applications in Acute Kidney Injury and Parkinson’s Disease. The purpose of this experiment is to improve the conversion rate of 7-ketocholic acid (7K-LCA) and the yield of ursodeoxycholic acid in aprotic solvents during electrochemical reduction process. Three aprotic solvents were investigated as electrolytes. 1,3-Dimethyl-2-imidazolidinone (DMI) has a stable five-membered ring structure, and 7K-LCA has undergone two nucleophilic reactions and “Walden” inversion, the 7K-LCK was stereoselectively reduced to UDCA. Hexamethylphosphoramide (HMPA) and 1,3-methyl-3,4,5,6-Tetrahydro-2(1*H*)-pyrimidinone (DMPU) can be attacked by chloride ions to produce by-products. Molecular orbital theory-based simulations were conducted to study the reducibility of three aprotic solvents [hexamethylphosphoramide (HMPA), 1,3-methyl-3,4,5,6-Tetrahydro-2(1*H*)-pyrimidinone (DMPU), and 1,3-Dimethyl-2-imidazolidinone (DMI)] in combination with experiments. Choose the best solvent based on the simulation results, the electrolysis reaction can be carried out by applying current and voltage when lithium chloride is used as electrolytes. Calculations using Materials Studio showed that Cu, Pb, Hg–Cu, and Ni exhibited the highest binding energies to the substrate in this system. Using Cu as the electrode when the solvent is a 1:1 mix of DMI and HMPA, the conversion rate of 7-ketocholic acid (could reach 98%, the yield of ursodeoxycholic acid was up to 80%. Under the same conditions, linear voltammetry was performed on the electrochemical workstation to study the electrolysis behavior, and the obtained results were consistent with the experiment.

**Graphical Abstract:**

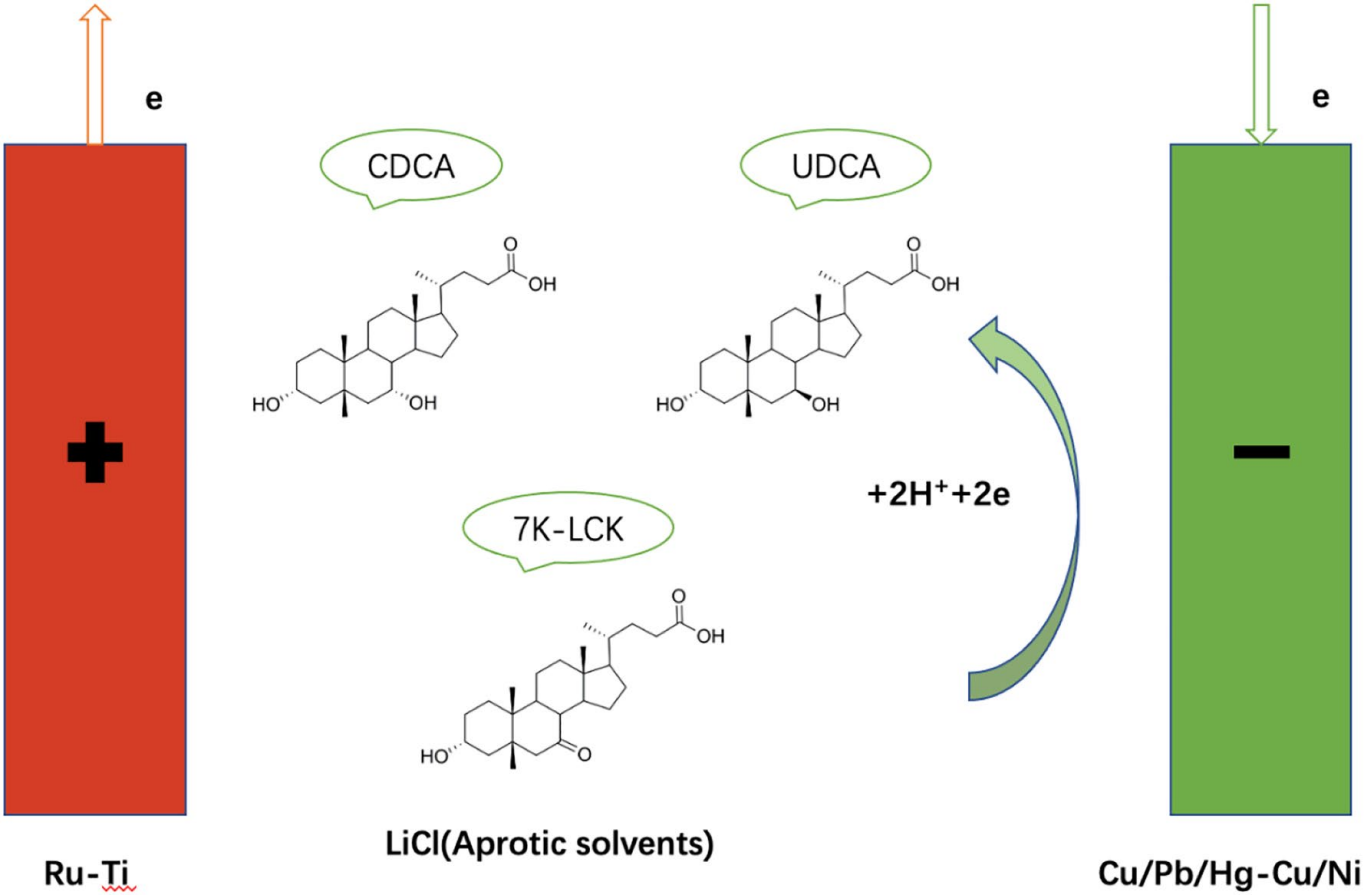

## Introduction

Ursodeoxycholic acid (UCDA) was first discovered by Hammarsten in polar bear bile in 1902 and named in the late 1910s. Ursodeoxycholic acid is a fundamental treatment drug for numerous hepatobiliary diseases that also has adjuvant therapeutic effects on certain cancers and neurological diseases, in addition, it also can be beneficial in neonatal jaundice needing phototherapy (Lazarus et al. 2022; Huang and Cao 2015; Xiaolei and Xuejun 2014; Liu et al. 2006; Santos et al. 2003). Since there is no side effect, ursodeoxycholic acid is also widely used in kidney injury (Acute Kidney Injury) and Parkinson’s Disease (Sathe et al. 2020; Qi et al. 2021; Luxenburger et al. 2023), has important clinical value. At the beginning, it was too cruel to extract ursodeoxycholic acid by killing bear bile or extracting bile duct drainage, so there were many methods to product ursodeoxycholic acid, and now many methods have been very mature.

**Fig. 1:**
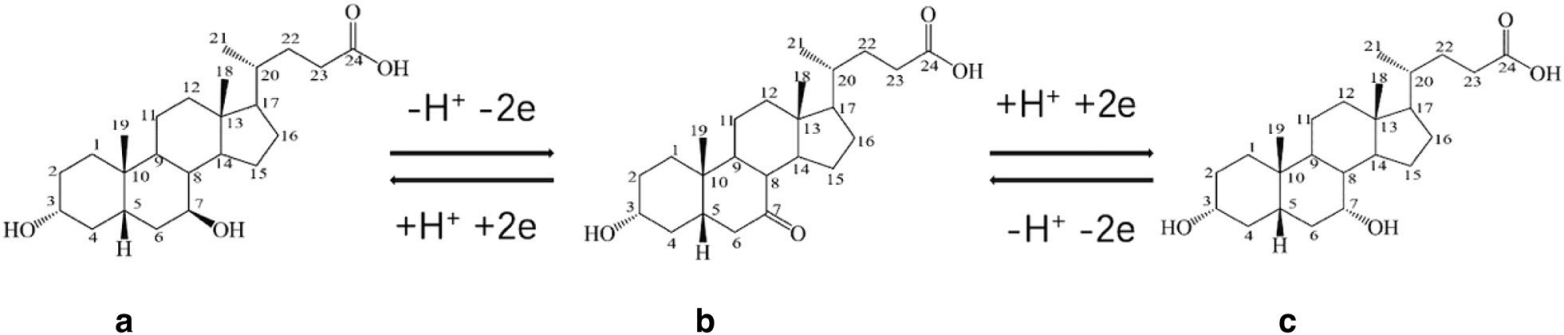
Chemical structure of UDCA (**a**), 7K-LCA (**b**) and CDCA (**c**)

As shown in Fig. 1, the structures of 7K-LCA, UDCA and CDCA are very similar. The only difference is that the positions of C7 are keto group and hydroxyl group, respectively. Since CDCA can be extracted in large quantities from cheap animals, such as chickens and geese, the hydroxyl group at the position of CDCAC7 is oxidized into keto group and becomes 7K-LCA first in previous studies. Then, restore 7K-LCA to UDCA. The most commonly used methods for UDCA synthesis are chemical, biological and electrochemical synthesis methods. The initial process is to selectively reduce 7K-LCA to UDCA using chemical methods, 7K-LCA is reduced to UDCA by adding catalyst in alcohol organic reagent under specific temperature and pressure, with the highest yield of 97% (Bharucha and Slemon xxxx; Hattori et al. xxxx). A novel synthetic route of producing ursodeoxycholic acid (UDCA) was synthesized from industrial plant materials recently. Ursodeoxycholic acid was synthesized in 9 steps through regional selective oxidation, Mistunobu reaction and Luche reduction with an overall yield of 38.6% (Chen et al. 2021). In 2022, a continuous flow selective hydrogenation method was developed for the preparation of ursodeoxycholic acid from Raney nickel, almost complete conversion, and a selectivity of 94.0% (Kim et al. 2023). Biosynthesis typically uses 7 beta-HSDH (7 beta-steroid dehydrogenases), using Rs7β-HSDH (NCBI accession no. WP_213611802.1) and an NAD+-dependent 7α-HSDH (7α-hydroxysteroid dehydrogenase), with O2/NOX and HCOO−/FDH (formate dehydrogenase) systems for the regeneration of cofactors (NAD+ and NADH), 25 mM chenodeoxycholic acid was completely converted to UDCA in one-pot two-step cascade, with an 80% isolated yield. In this paper, the electrochemical method is used to prepare ursodeoxycholic acid, which is a relatively simple and safe process that does not require harsh reaction conditions and complex reaction steps (Yang et al. 2023).

In 2011, Cole et al. (xxxx) found that the introduction of a carbonyl compound into a split electrochemical cell containing electrolytes and the cathode reduced the carbonyl compound to at least one aldehyde compound; Aldehydes are altered by adjusting (i) the anode electrode material, (ii) the electrolyte, (iii) the heterocyclic catalyst, (iv) the pH, and/or (v) the potential. Silvia Mena et al. (2021) found that in the experiment of electrochemical reduction of CO_2_, cyclic voltammetry and molecular dynamics simulation can be used to quickly determine the parameters and predict the most suitable electrode materials and solvents.

The solvents often used in electrochemical reduction are ionic liquids, methanol solutions, and organic aprotic solvents (Konig et al. 2021). The relatively low cost of organic aprotic solvents, wide “electrochemical windows,” and high solubility make them a promising medium in the electrochemical conversion of 7K-LCA (Kuntyi et al. 2022), in addition, the simplicity of the design of the diaphragm-free electrolyzer, high conversion speed, and high Faradaic efficiency of products make this method promising. Adding hexamethylphosphoric triamide (HMPA) to the reduction reaction can prevent the carbonyl group from being reduced to a stable ketone radical anion. In addition to enhancing the stereoreducibility of the reaction, reactivity studies have shown that it also has effective proton transfer capabilities (Sandipan and Shmaryahu 2014). According to research, it was found that 1,3-methyl-3,4,5,6-tetrahydro-2(1*H*)-pyrimidinone (DMPU) also has a certain catalytic ability in the reduction reaction (Khan et al. 2013), and reduce the energy required for the reaction to occur, apart from this, DMPU is considered to be the best basis for promoting intramolecular hydride delivery (Li et al. 2018). When DMPU and HMPA are used as electrolyte, CDCA is synthesized stereoselectively. 1,3-Dimethyl-2-imidazolidinone (DMI) is also a special solvent that promotes reduction and can be used as an electrolyte for stereoselective synthesis of UDCA (Tsugio et al. 2013), Because the five-membered imidazole ring structure of DMI is very stable, chloride ion acts as a nucleophile to attack 7K-LCA. Then, hydroxide ion continues to attack and replace chloride ion, undergoing two “Walden inversions” and then the 7K-LCA can be stereoselectively reduced to UDCA without CDCA as a by-product.

Molecular simulation technology can calculate the diffusion behavior through binding energy of the target molecule and the metal surface in the solution medium. In 2014, Guo et al. studied the three molecules of 4-chloro-acetophenone-*O*-1′-(1′0.3′0.4′-triazolyl)-metheneoxime (CATM), 4-fluoro-acetophenone-*O*-1′-(1′0.3′0.4′-triazolyl)-metheneoxime (FATM), and 3,4-dichloro-acetophenone-*O*-1′41′0.3′0.4′-triazolyl)-metheneoxime (DATM) through a systematic molecular simulation method, and the order of their absolute value of adsorption energy on the Fe(110) surface is consistent with the corrosion inhibition efficiency value obtained from the experiment. 7K-LCA loses two electrons and two protons on the surface of the ruthenium–titanium anode electrode and oxidizes into 3,7 Ketolithocholic acid, and then obtains two electrons and two protons on the surface of the cathode, which undergoes a reduction reaction to generate products.

In the previous work (Shen et al. 2021; Huang and Cao 2015), 7K-LCA, an important intermediate in UDCA synthesis, was synthesized by electrooxidation using methanol as solvent in two-cell electrolytic cell, the conversion rate of 7K-LCA reached 64.2%, and the yield of UDCA was 44.5%. Then, the electrolytic cell was changed into a single cell electrolytic cell, and the aprotic solvent was used as the electrolyte for electrochemical reduction, and the yield was only 34.9%. The purpose of this paper is to improve the yield of UDCA using linear voltammetry and molecular simulation on the basis of previous experiments. In this study, the C-7 carbonyl group of 7K-LCA was reduced to 7β-hydroxyl group on the surface of cathode electrode in the electrolyte composed of aprotic solvent in a single cell electrolytic cell with LiCl as electrolyte, so as to stereoselectively reduce 7-ketocholic acid to ursodeoxycholic acid. The yield can be changed by adjusting (i) the cathode material, (ii) the rotational speed, (iii) the aprotic solvent ratio, and (iv) the amount of electrolyte.

## Experimental

### Chemical reagent

Three aprotic solvents, hexamethylphosphoramide (HMPA, 98%), 1,3-dimethyl-3,4, and 5,6-tetrahydro-2(1*H*)-Pyrimidone (DMPU, 99%), dimethyl-2-imidazolidinone (DMI, 98%) purchased from Shanghai Macleans Biochemical Technology Co., Ltd. Anhydrous lithium chloride (Shanghai Aladdin Biochemical Technology Co., Ltd, 99%); 7-ketolithocholic acid (7K-LCA, Anhui Kebao Bioengineering Co., Ltd., 99%); Ursodeoxycholic acid (UDCA, 99%) and Chenodeoxycholic acid (CDCA, 99%) standard products purchased from Shanghai Maclean Biochemical Technology Co., Ltd.; mercury (Hg, 99%) from Shanghai Aladdin Biochemical Technology Co., Ltd.; Sinopharm Chemical Reagent Co., Ltd. purchased methanol, alcohol, acetonitrile, etc.

### Instrumentation

The linear sweep volt-ampere curve (LSV) was measured with Cyclic voltameter with an AUTOLAB PGSTAT30 Electrochemical Workstation (Eco Chemie B.V., The Netherlands). The electrolyzer (sealed three-electrode electrolytic cell) was purchased from China Instrument No. 1 Store. The various metal wires used are purchased from Yudingda Metal, and the mercury-plated copper wire is self-made. Saturated calomel electrode (SCE, Shanghai Leici shop) is used as reference electrode (RE), and constant current electrolysis adopts HYD250-1 DC stabilized power supply (Shanghai Huyi Technology Co., Ltd.). Copper, lead, nickel, mercury and copper (except for mercury on copper are plated by themselves, and the rest were purchased from Yudingda Metal) as cathode materials, ruthenium and titanium as anode materials, and the whole experiment was carried out in an anhydrous state. The HPLC instrument (LC-20A) was from Shimadzu Corporation, Kyoto, Japan. The single tank reaction device is shown in graphical abstract.

### Methods

Blowed nitrogen gas into each aprotic solvent liquid containing 0.25 g of anhydrous lithium chloride to remove oxygen which affects the subsequent test. Aprotic solvents used were hexamethylphosphoramide (HMPA), 1,3-dimethyl-3,4,5,6-tetrahydro-2(1*H*)-pyrimidinone (DMPU), dimethyl-2-imidazolidinone (DMI), and a mixture of HMPA and DMI. Sealed the electrolytic cell, installed the electrode to be tested, and performed the LSV test in the electrolyte. After the test, added 1.0 g 7K-LCA to each different electrolyte, repeat the operation of nitrogen gas and then seal, continued the LSV test, compared the linear sweep voltammograms obtained under different scanning rates or under different electrodes.

Preparation of standard samples: prepare a water–acetone mixed solution with a volume ratio of 1:9, then weigh 0.01 g of UDCA, CDCA and 7K-LCA standards, and dissolve them in 1 mL of water–acetone mixed solution to obtain a concentration of 10 mg/mL standard pure product solution. Preparation of product samples: weigh 0.01 g of the product obtained after separation and drying at the end of electrolysis, and dissolve it in 1 mL of water–acetone mixture to make the concentration 10 mg/mL. The *R*^2^ values of all standard curves are greater than 0.999.

Dissolved 0.1 M anhydrous LiCl in 60 mL different aprotic solvents, respectively, added 1.0 g 7K-LCA after completely dissolving, in constant current electrolysis, direct current was used, Ru/Ti was the anode, Cu, Pb, Hg–Cu, Ni was the cathode, and a 100 mL beaker was used as the electrolytic cell, installed the anode and cathode, and then connected to the electrolyzer for constant current electrolysis, setting different currents. The total electrolysis time was 24 h, and samples were taken every 2 h. After the electrolysis, the conversion rate of 7K-LCA and the yield of UDCA and CDCA were detected by high performance liquid chromatography. The aprotic solvents were HMPA, DMPU, DMI, and a mixture of DMI and HMPA in different proportions. Added different LiCl concentrations can also increase the ion conductivity. Added 0.25 g, 0.5 g, and 0.75 g (0.1 M, 0.2 M, 0.3 M) LiCl, and then 1 g of 7K-LCA as a raw material was added to the electrolyte, using the electrolyzer to set different currents, the electrolysis time was 24 h.

Used Gauss view to draw the DMI, DMPU and HMPA models, and further used the B3LYP method in DFT to optimize the structure at the level of the 6-311g** unit and calculated the highest occupied molecular orbital (HOMO) and lowest of the corresponding molecules. Empty molecular orbital (LUMO), dipole moment and energy gap (∆*E* = *E*_LUMO_ − *E*_HOMO_). Using a mixture of DMI and HMPA as the electrolyte, the adsorption behavior of 7-ketolithocholic acid in the electrolyte on the surface of the electrode was studied using the Forcite module in the MaterialStudio software. The research objects were 5 layers of Cu(001) surface, 5 layers of Pb(001) surface, 5 layers of Ni(001) surface and 1 molecule of 7 k, 12 molecules of DMI and 10 molecules of HMPA. Molecular dynamics (MD) simulation used COMPASS force field and a typical NVT ensemble, with a time step of 1.0 fs and a simulation time of 500 ps. The interaction energy (*E*_interat_) between the compound and the metal substrate were calculated according to the formula *E*_binding_ = *E*_tot_ − (*E*_sub_ + *E*_inh_), where *E*_interact_ is the total energy of the entire system, *E*_sub_ is the energy of the copper substrate and all H20 molecules, which is the energy of 7K-LCK.

### Analytical methods

The electrolyte is an aprotic solvent. Different experimental conditions were changed for comparison. The process lasts for 24 h, and samples are taken continuously during the reaction. After the reaction, the products were tested by high performance liquid chromatography. The electrolysis is completed in 24 h. After sampling, add the electrolyte to 400 mL of water, adjust the pH with HCl (1 mol), and filtrate the product after precipitation. Place the product in a 60 °C vacuum oven (Shanghai Yiheng Scientific Instrument Co., Ltd.) until it is completely dried.

The samples obtained during the 24-h electrolysis process were quantitatively analyzed by high performance liquid chromatography using a C-18 column. The mobile phase is acetonitrile/phosphate buffer (pH 2–3) with a volume ratio of 1:1. The HPLC samples was injected at 1.0 mL/min at 40 degrees Celsius, and the UV detection wavelength was 208 nm. Use 10% isopropanol to clean the syringe in the SIL-20A autosampler (Shimadzu Corporation, Kyoto, Japan). The retention times of 7-ketolithocholic acid, ursodeoxycholic acid and chenodeoxycholic acid are 11.5–12.5, 9.4–9.8, and 18–19 min, respectively.

1 $${\text{The}}\;{\text{conversion}}\;{\text{rate}}\;{\text{of}}\;7{\text{K - LCA}}\;{\text{is}}\;C = {\text{Mt}}/{\text{M}}0 \times 100\% ;$$

Mt is the difference value between the total concentration of 7K-LCA before and after reaction; M0 is the total concentration of 7K-LCA before reaction.

2 $${\text{The}}\;{\text{total}}\;{\text{yield}}\;{\text{of}}\;{\text{UDCA}}\;{\text{is}}\;{\text{Yu}} = {\text{Ua}}/{\text{Ut}} \times 100\% ;$$

Ua is the concentration of UDCA after electrolysis, and Ut is the total concentration of 7K-LCA.

3 $${\text{The}}\;{\text{yield}}\;{\text{of}}\;{\text{CDCA}}\;{\text{is}}\;{\text{Yc}} = {\text{Ca}}/{\text{Ct}} \times 100\% ;$$

Ca is the concentration of CDCA before electrolysis, and Ct is equal to the total concentration of 7K-LCA before electrolysis.

At last, thin-layer chromatography can be used for qualitative analysis of the product and further determine the product.

## Results and discussion

### Influence of linear voltammetry scanning

**Fig. 2:**
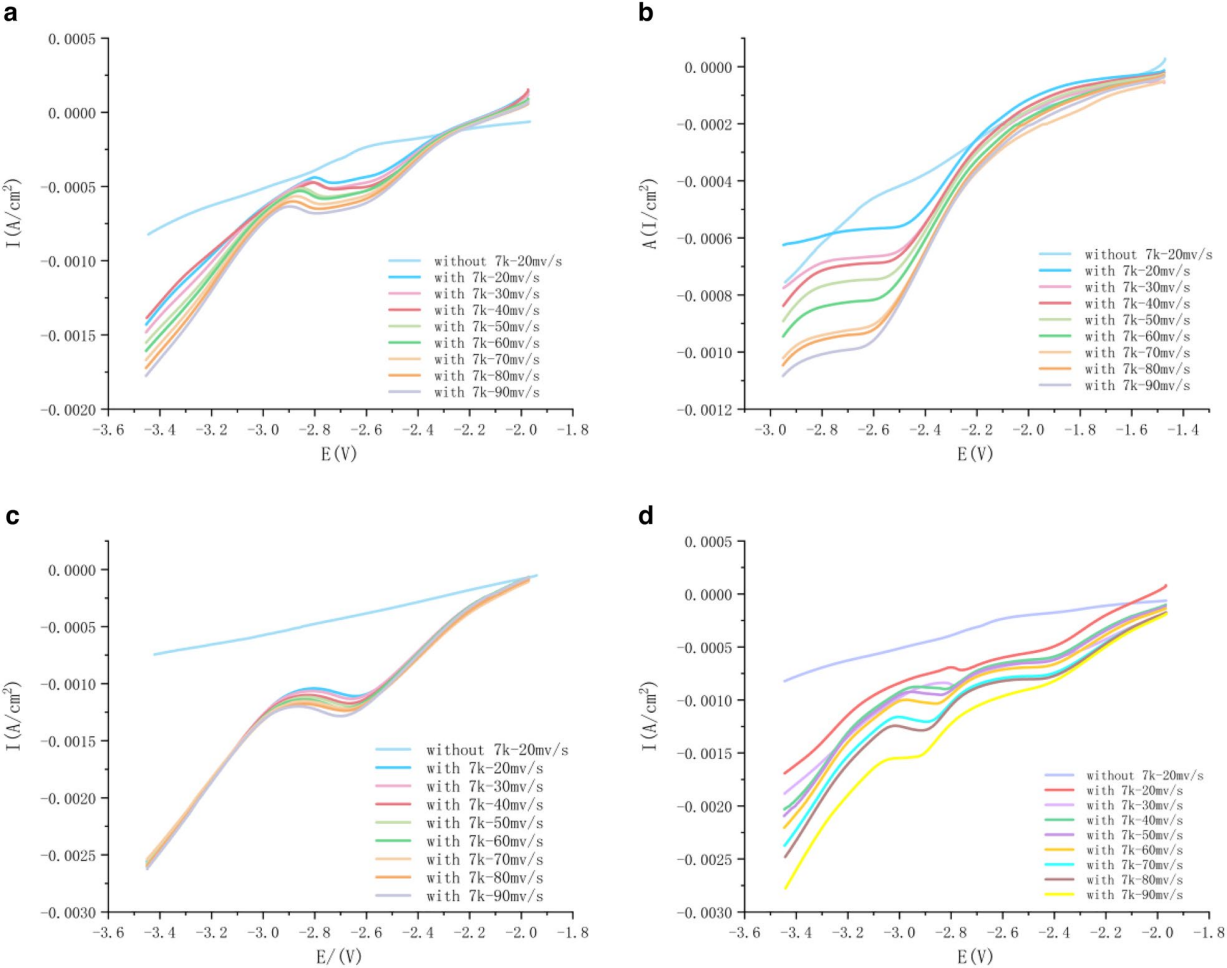
LSV of without 7K-LCK at 10mv/s of scan rate and with 7K-LCA at different scan rate(**a**:DMI, **b**:DMPU, **c**:HMPA, **d**:DMI&HMPA)

It can be seen from Fig. 2 that the LSV results of 7K-LCA in different electrolytes are significantly different. No matter what the electrolyte is, when there is no 7K-LCK, there is no reduction peak in the linear voltammetry curve. When the electrolyte contains 7K-LCK, an obvious reduction peak will appear in the LSV curve. Then, set different scanning speeds to perform linear volt-ampere scanning. It can be seen from the following figure that as the scanning rate increases linearly, the reduction peak *E*_p_ of 7K-LCA showed a regular negative shift, and the peak current *I*_p_ gradually tends to increase regularly.

**Fig. 3:**
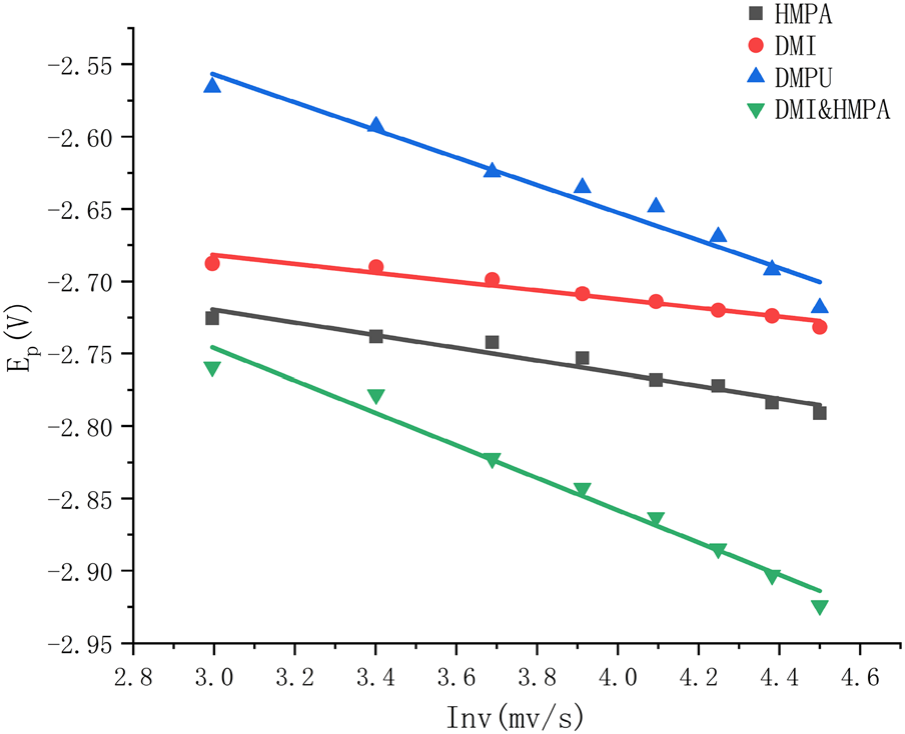
Plot of E_p_ versus Inv for HMPA、DMPU、DMI and DMI&HMPA systems

As shown in Fig. 3, taking the logarithm of the increasing scanning speed as the abscissa, and the reduction peak potential as the ordinate, the mixed curves of DMI, HMPA, DMPU, DMI and HMPA are drawn into graphs. *E*_p_ and Inv in each electrolyte have a linear relationship, indicating that the reduction reaction of 7K-LCK in these four electrolytes is irreversible. In the experiment, once the substrate is reduced to UDCA or CDCA, it is impossible to be oxidized to 7K-LCK again.

Using the square root of the set scan rate as the abscissa and the reduction current *I*_p_ as the ordinate, the curves *I*_p_ − *v*^1/2^ of four different aprotic solvent systems are made. As shown in the figure, the correlation coefficients of the four curves are 0.9939, 0.9970, 0.9983 and 0.9890, indicating that *I*_p_ and *v*^1/2^ present a good linear relationship, indicating that the irreversible reduction reaction carried out in the experiment is affected by diffusion. Considering that the reduction reaction is affected by diffusion, the rotor was put into the electrolyte and the system was placed on the magnetic stirrer during the experiment, and the different speeds were adjusted to compare the results.

### Effect of different solvents

According to the frontier orbital theory, the reactivity of a molecule is related to its highest occupied molecular orbital and the lowest empty molecular orbital. It is generally believed that a lower ELumo value indicates that the molecule has a stronger ability to act as an electron acceptor, while a high EHomo value means that the molecule has a stronger electron donating ability. Therefore, the energy gap reflects the chemical stability of organic molecules, and the lower the Δ*E* value, the easier it is for organic molecules to be adsorbed on the metal surface. The higher the dipole moment, the stronger the ability of organic molecules to adsorb on the metal surface.

**Fig. 4:**
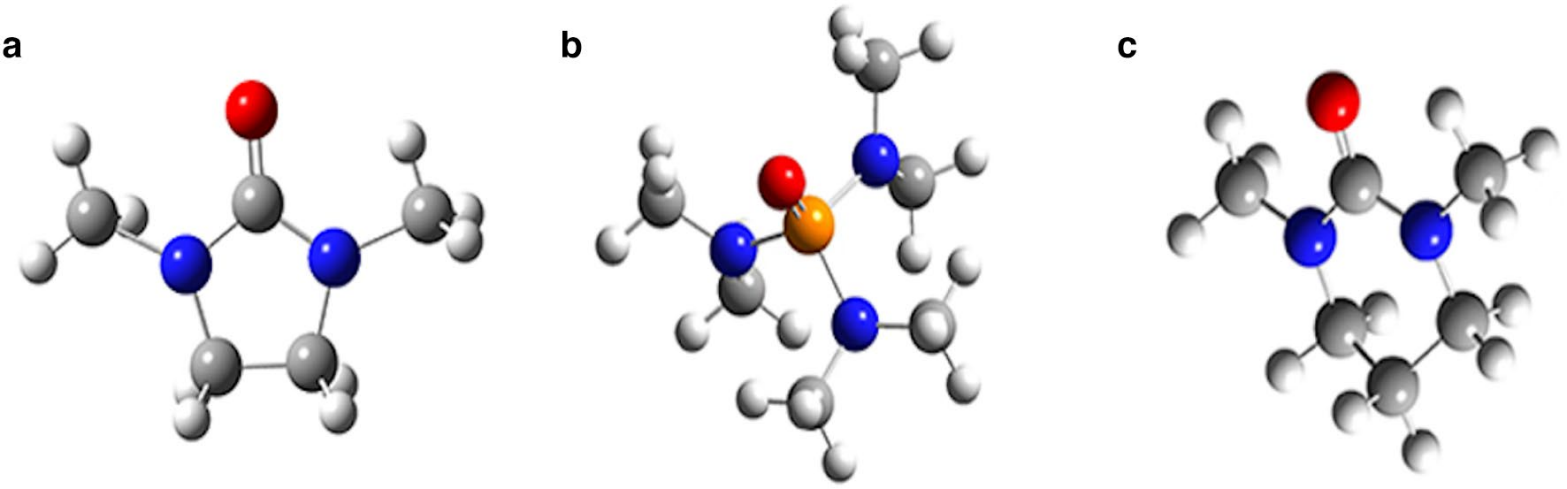
Gaussian model of different solvent molecules(**a**:DMI, **b**:HMPA, **c**:DMPU)

The molecular models of DMI, HMPA and DMPU drawn and optimized by Gaussian software are shown in Fig. 4. It can be seen from Table 1 that the ∆*E* value in descending order are 8.679891, 6.662768, 6.671415, when DMI, DMPU and HMPA were used as solvents. In addition, the dipole moments in descending order are 4.0963, 3.7477, 3.5009, when DMPU, HMPA and DMI were used as solvents, which indicates that DMPU has the strongest adsorption capacity on the surface of the metal electrode, followed by HMPA. The last is DMI. The stronger the adsorption capacity of organic molecules, the stronger the binding of the substrate molecules to the metal electrode.

**Table 1:** Parameters calculated by DFT for different aprotic solvent molecules

	*E*_LUMO_ (eV)	*E*_HOMO_ (eV)	Δ*E* (eV)	*µ* (Debye)
DMI	2.181334	− 6.498557	8.679891	3.5009
HMPA	0.610348	− 6.061067	6.671415	3.7477
DMPU	0.405026	− 6.257742	6.662768	4.0963

### Effect of different concentrations of supporting electrolyte

**Fig. 5:**
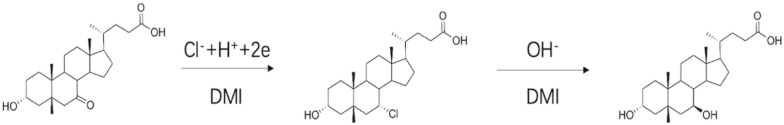
Schematic diagram of reaction mechanism in DMI

The product comparison of three different aprotic solvents as electrolytes in Table 2 shows that when only DMI is the solvent, the yield of UDCA is the highest and no by-product CDCA is generated. The reasons are as follows: the five-membered imidazole ring structure of DMI is stable, as shown in Fig. 5, 7K-LCA undergoes two nucleophilic and “Walden inversions”. The chloride ion attacks the hydroxyl group at the 7-position, which can make the chiral center carbon of 7K-LCK occur. The configuration is reversed, and the hydroxide ion in solution further attacks the chloride ion, thereby stereoselectively reducing 7K-LCA to UDCA. The structures of HMPA and DMPU are relatively unstable, and chloride ions can directly attack them. 7K-LCA obtains hydrogen ions on the cathode electrode under the action of electric current and directly undergoes a reduction reaction. Compared with UDCA, CDCA has less steric hindrance, so part of 7K-LCA stereoselectively reduced to CDCA.

**Table 2:** Effects of electrolyte addition in different aprotic solvents on electrochemical reduction reaction

Solvents	LiCl (%)	*C* (%)	*Y*_u_ (%)	*Y*_c_ (%)
HMPA	0.1 M	94.2	10.7	21.1
	0.2 M	98.5	12.3	24.8
	0.3 M	–	–	–
DMPU	0.1 M	39.3	8.2	9.7
	0.2 M	50.2	11.8	12.4
	0.3 M	62.4	11.3	19.6
DMI	0.1 M	34	22	–
	0.2 M	43.6	28.4	–
	0.3 M	30.2	18.2	–

Theoretically, the greater the amount of electrolyte in the electrolysis system, the better the conductivity, and, therefore, the better the effect of substrate electrolysis. If more electrolyte is added to the electrolysis system, at the same time, a smaller voltage value can be set. It can be seen from Table 2 that adding different amounts of electrolyte to DMI, HMPA, and DMPU will affect the electrochemical reduction results of 7K-LCK, and the best LiCl concentration is 0.2 M. In the DMPU system, although the substrate conversion rate is becoming higher with the increase of the electrolyte concentration, the products yield was not raised significantly, and the by-product output was increased. In the HMPA system, 0.3 M LiCl cannot be dissolved, and the overall conversion rate of 7K-LCK is very high. When the electrolyte concentration is high, the electrolysis rate will be accelerated, but if the electrolyte concentration is too high, the current will increase, the thermal effect can cause the honeycomb expansion of sample components, and the electrolytic efficiency will be worse, and the effect will be lower. Among the three electrolysis systems, the DMI system can obtain the largest amount of UDCA, and the effect was best when 0.2 M LiCl is added. At this time, the yield of UDCA is 28.4%, and by-product CDCA was not produced.

### Effect of the ratio of HMPA to DMI

The yields of UDCA and CDCA produced by electrochemical reaction in aprotic solvent are not equal to the conversion rate of 7K-LCK, and intermediate products were generated during the reaction. The initial conjecture is 3,7-ketolithocholic acid, UDCA loses hydrogen ions on the anode, the –OH at the 3rd position and the –OH at the 7th position can be oxidized to become =O, and 3,7-ketolithocholic acid can be produced, Hydrogen ions are obtained at the cathode to generate UDCA and CDCA, which are not completely reduced. In addition, in the molecular simulation content of this chapter, it is known that the binding force between DMI and HMPA and the metal electrode surface is relatively small in the three aprotic solvents. The essence of this experiment is that the substrate undergoes a reduction reaction on the cathode electrode, so these two solvents relatively does not affect the reaction of the substrate 7K-LCK with the metal electrode. Based on the above content, DMI and HMPA are mixed as the electrolyte, 0.2 M electrolyte is added, and the two aprotic solvents are mixed in different ratios for experimental comparison. As shown in Table 3, with the increase of the HMPA solvent content in the electrolyte, DMI solvent content decreases, the conversion rate of 7K-LCK is getting higher and higher, the yield of by-product CDCA is also getting higher and higher, the yield of target product UDCA continues to increase due to the increase of the conversion rate of substrate 7K-LCK, the optimal mixing ratio of DMI and HMPA solvent is 1:1. At this time, the conversion rate of substrate 7K-LCK was 94.7%, the yield of target product UDCA was 68.1% and the yield of by-product CDCA was 25.5%.

**Table 3:** Influence of the ratio of DMI to HMPA on the stereoselective electroreduction of 7K-LCA

Solvents	Conversion of 7K-LCA (%)	Yield of UDCA (%)	Yield of CDCA (%)
10%HMPA + 90%DMI	73.1 ± 1.4	28.3 ± 1.6	13.5 ± 0.5
15%HMPA + 85%DMI	82.2 ± 0.3	31.6 ± 0.5	16.4 ± 0.3
20%HMPA + 80%DMI	86.7 ± 0.4	34.3 ± 0.2	20.7 ± 0.2
30%HMPA + 70%DMI	93.8 ± 0.2	39.4 ± 0.4	22.4 ± 0.3
40%HMPA + 60%DMI	93.9 ± 0.3	50.4 ± 0.5	23.1 ± 0.4
50%HMPA + 50%DMI	94 ± 0.7	67.8 ± 0.3	25.3 ± 0.2
60%HMPA + 40%DMI	92 ± 0.3	58.9 ± 0.4	27 ± 0.5
70%HMPA + 30%DMI	95 ± 1.4	58.6 ± 0.8	28.4 ± 0.9
80%HMPA + 20%DMI	96.7 ± 0.7	58.5 ± 0.7	30 ± 0.7
90%HMPA + 10%DMI	97.7 ± 0.5	47 ± 0.6	35.6 ± 0.5

### Effect of different mixing speeds

**Fig. 6:**
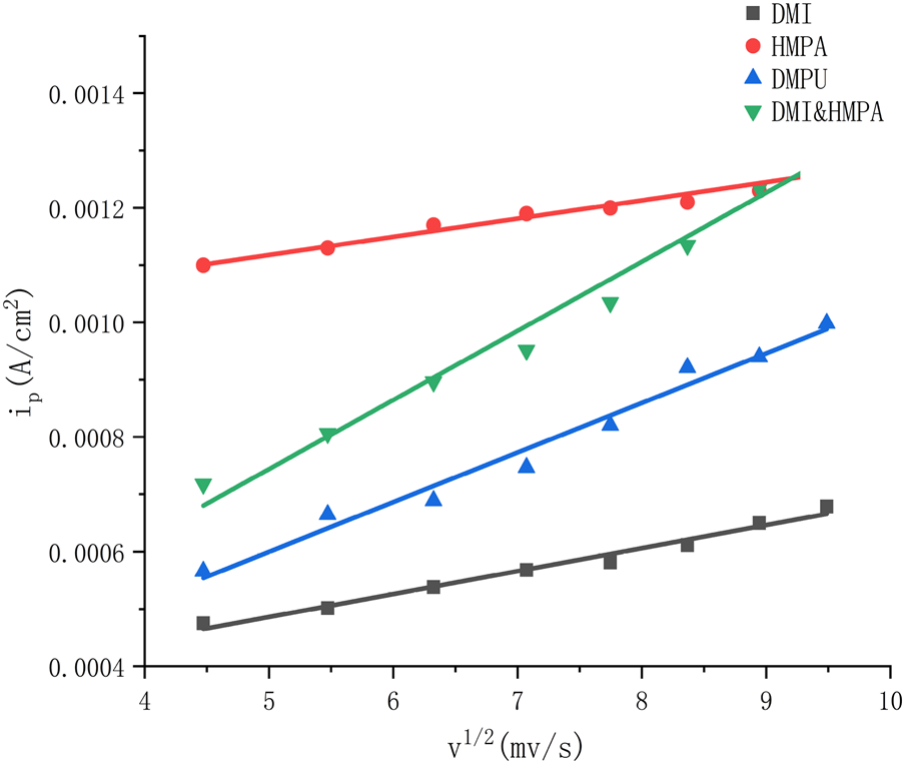
Plot *I*_p_ versus *v*^1/2^ for DMI, HMPA, DMPU and DMI&HMPA systems

**Fig. 7:**
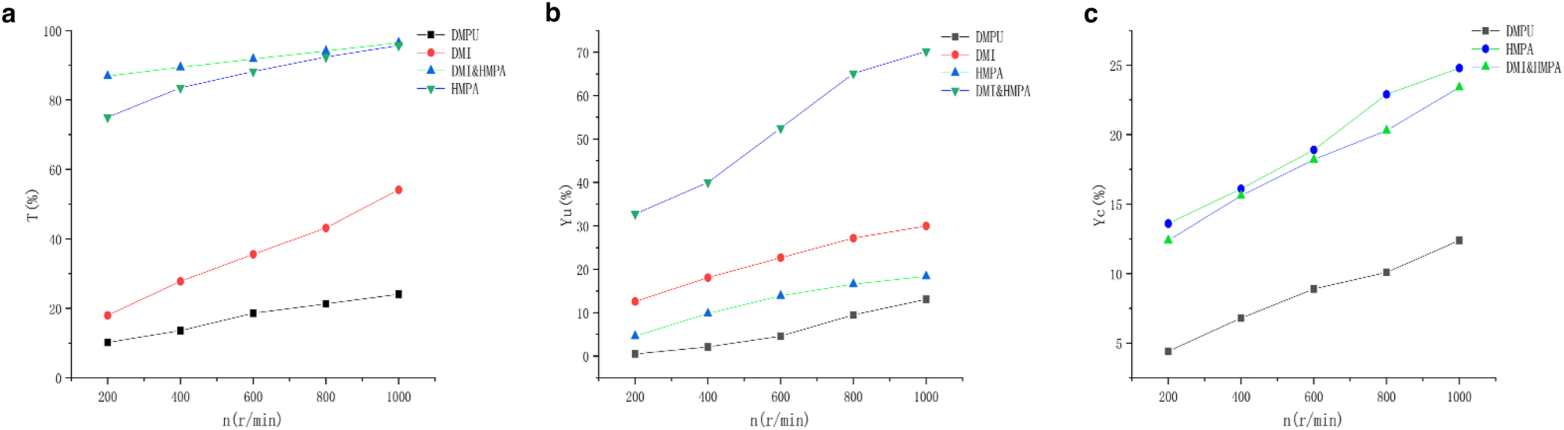
Influence of different speeds on electrochemical reduction (**a** 7K-LCA, **b** UDCA, **c** CDCA)

According to the linear voltammetry curve in Fig. 6, by making the curve obtained by fitting the *I*_p_ and *v*^1/2^ scatter diagram, it can be seen that the linear relationship between *I*_p_ and *v*^1/2^ is very good, then it can be known that the reduction reaction is controlled by diffusion. In the reaction of electrochemical reduction of CO_2_ to CH_4_, stirring speed were together found to be proportional to the rate of CH4 production (Hashiba et al. 2016). Therefore, in the electrolysis process of DMI, DMPU, HMPA and the mixed solution of DMI and HMPA as electrolyte, the rotor was added to set different rotational speeds, and the results are shown in Fig. 7. After the reduction reaction of the four systems, within 1000 r/min, with the increase of rotation speed, the conversion rate of 7K-LCK gradually increased, and the yield of product UDCA also gradually increased, but the yield of by-product CDCA also showed an overall upward trend.

And it can be clearly seen that the conversion rate of substrate 7K-LCK and the yield of UDCA are the highest in the HMPA and DMI mixed solution electrolysis system, reaching 94.8% and 70% when the rotation speed is 1000 r/min.

### Effect of electrode materials on electroreduction

It can be seen from the above that the type of solvent, the amount of electrolyte, and the rotational speed during electrolysis all have an effect on the electrochemical reduction of the substrate 7K-LCK. Since the electrochemical reduction reaction occurs on the surface of the cathode electrode, the influence of different metals as the cathode electrode on the electrolysis reaction should be continuously explored. In an aprotic solvent mixed with 50% HMPA and 50% DMI, 0.2 mol of LiCl was added as the electrolyte, and four metal sheets of Cu, Hg–Cu, Pb and Ni were used as the cathode electrode for electrolysis. When Cu is used as the cathode, the yield of UDCA is the highest, reaching 74.3%, while the by-product CDCA is the least, only 16.8%. It can be seen that the Cu electrode is the best electrode.

**Fig. 8:**
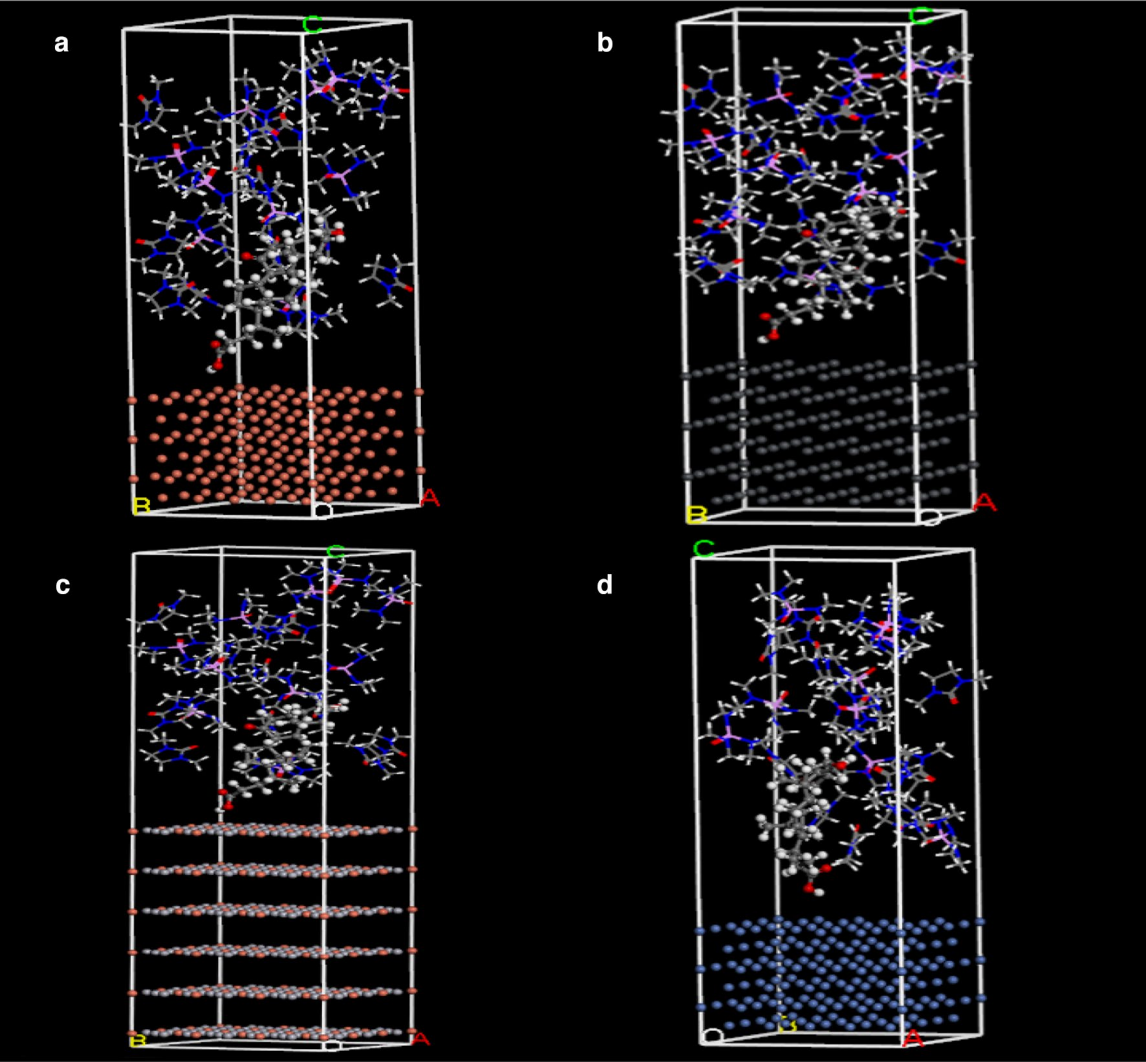
Materials studio model of electrolytic system with different electrodes (**a** Cu, **b** Pb, **c** Hg–Cu, **d** Ni)

Molecular dynamics (MD) simulation can explore the change of motion state of particles in a certain statistical mechanics system as time progresses to obtain the physical and chemical properties of the system. MD simulation can find the interaction between molecules and metal electrodes. This experiment investigates the 7K electro-reduction results under different electrodes. Since the reaction is carried out on the electrode surface, electrons are finally obtained from the cathode electrode to generate the final product. The simulation can calculate the binding energy of 7K-LCK molecules with different metal surfaces. According to the method, the model was built with Materials Studio software, as shown in Fig. 8, and the 7K-LCK was calculated in 50% DMI and 50% HMPA solvents and different electrodes.

Binding energies, as shown in Table 5, the calculated binding energies from small to large are Ni, Hg–Cu, Pb, Cu, which are − 49.353789, − 65.204288, − 69.127444, − 124.32943 kcal/mol, respectively, binding to the substrate 7K-LCK in the system. The biggest one is the copper electrode. As shown in Table 4, four kinds of metals were used as cathode electrodes. When Cu was used as cathode electrode, the 7K-LCK conversion rate and UDCA yield were the highest. They are 74.3% and 16.8%, respectively, which are consistent with the simulation results, indicating that the binding energy between the substrate and the electrode also affects the experimental results (Table 5).

**Table 4:** The influence of electrodes on electrochemical reduction (a: Cu, b: Pb, c: Hg–Cu, d: Ni)

Electrode	Conversion of 7K-LCA (%)	Yield of UDCA (%)	Yield of CDCA (%)
Cu	91	74.3	16.8
Pb	94	67.8	20.3
Hg–Cu	91	64.6	22.7
Ni	93.7	62.9	25.7

**Table 5:** Binding energy of substrate and different electrodes in molecular simulation

Electrode	*E*			
	*E*_inh_ (kcal/mol)	*E*_sub_ (kcal/mol)	*E*_tot_ (kcal/mol)	*E*_bunding_ (kcal/mol)
Cu layer	80.230448	556.780136	512.681154	− 124.32943
Pb layer	42.743639	467.791758	441.407953	− 69.127444
Ni layer	42.110363	514.465015	507.221589	− 49.353789
Hg–Cu layer	42.710252	491.668338	469.174302	− 65.204288

## Conclusion

With ruthenium titanium as the anode, lead as the cathode, LiCl as the electrolyte, and the three aprotic solvents as the system, the effect is best when 0.2 M LiCl is added. In the HMPA solvent, the conversion rate of 7K-LCA is up to 98.5%. In the DMI solvent, the conversion rate of 7K-LCA reached 43.6% at the highest, due to the stable five-membered ring structure of the DMI molecule, 7K-LCA has undergone two nucleophilic and “Walden inversions”, thus stereoselectively 7K-LCA is reduced to UDCA, no by-product CDCA is produced. DFT calculations were performed to study different aprotic solvent molecules, and it was found that the adsorption capacity of HMPA and DMI solvent and metal electrode surface is relatively weak.

Mixed HMAP and DMI aprotic solvent to form a composite electrolyte, and explore the optimal process conditions for electrochemical reduction of 7K-LCK. In the electrolysis system with different aprotic solvents as electrolytes, the optimal concentration of electrolyte in the electrochemical reduction of 7K-LCK experiment is 0.2 M. On this basis, when the rotation speed was 1000 r/min, the 7K-LCK substrate was almost completely converted into the target product UDCA, reaching the highest value of 68.1%, and the yield of the by-product CDCA was 25.5%. When Cu is the cathode, the conversion of 7K-LCK was 91%, the yield of UDCA was 74.3%, and the yield of CDCA was 16.8%. NVT calculations were performed to study binding energies of metal electrodes to substrates in electrolysis systems, it is found that the binding energy of Cu to the substrate in the system is indeed the highest.

## Data Availability

All data generated or analysed during this study are included in this published article.
